# Choroidal thickness and high myopia: a cross-sectional study and meta-analysis

**DOI:** 10.1186/s12886-015-0059-2

**Published:** 2015-07-03

**Authors:** Shiming Wang, Yong Wang, Xiaoming Gao, Nana Qian, Youer Zhuo

**Affiliations:** Ningbo Aier Guangming Eye Hospital, 8 Huancheng West Road, 315020 Ningbo, China; Wuhan Aier Eye Hospital, Wuhan, China

**Keywords:** Choroidal thickness, High myopia, Optical coherence tomography

## Abstract

**Background:**

The purpose of this study was to examine the choroidal thickness of patients with high myopia using enhanced depth imaging optical coherence tomography (EDI-OCT) and compare them with healthy subjects.

**Methods:**

We first conducted a cross-sectional study and then performed a meta-analysis to address this issue further. Using enhanced depth imaging optical coherence tomography (EDI-OCT), the macular choroidal thickness of high myopic eyes and normal control eyes were measured and compared at each location. Univariate and multivariate linear regression analyses were performed to assess the association between choroidal thickness and clinical factors such as axial length (AL), spherical equivalent (SE), and central corneal thickness. In the high myopic eyes, subgroup analysis of macular choroidal thickness was performed in eyes with or without lacquer cracks and choroidal neovascularization (CNV). The meta-analyses were conducted using the Stata software package.

**Results:**

The high myopic eyes had a thinner choroid than the control eyes at all macular locations (all *P* < 0.001). Multivariable linear regression analysis showed that the subfoveal choroidal thickness (SFCT) was not significantly thinner in association with the diagnosis. Subgroup analysis showed that the high myopia with CNV and with lacquer cracks had a significantly thinner choroid than without CNV or lacquer crack eyes. The result of our cross-sectional study is consistent with the results of the further meta-analysis with the pooled weighted mean difference (WMD) of −116.30 μm (95 % CI: −145.68, −86.92) for SFCT.

**Conclusions:**

The present study, along with the comprehensive meta-analysis, indicated that in the Chinese population, the choroidal thickness in high myopic eyes was thinner than that of normal control eyes, even across different subgroups. This might be secondary to the longer AL but it is not an independent factor. The presence of CNV and of lacquer cracks is associated strongly with eyes with thinner macular choroids.

## Background

High myopia is one of the main causes of visual impairment worldwide [1]. It has been reported that about 1 % of the population suffers from this disease [2]. High myopia is always accompanied by pathological structural changes, such as axial elongation, posterior scleral staphyloma, lacquer crack formation, thinning of the retina and chorioid, and choroidal neovascularization (CNV) [3–5]. Excessive axial elongation of the eyeball is thought to be one of the main causes of the ocular complications mentioned above. Among these complications, chorioretinal atrophy, augmented by choroidal thinning, can lead to photoreceptor cell death, which results in the consequent loss of central visual function. Another notable complication threatening visual function is CNV [4]. Myopic CNV develops into secondary chorioretinal central atrophy and leads to central scotoma [6].

As we all know, in high myopic eyes, the earliest changes begin in the choroid [7]; recent interest has focused on the choroid as an important structure involved in the pathophysiology of high myopia [8]. Choroidal thickness may be an important parameter in studying the pathogenesis of high myopia. With the enhanced depth imaging (EDI) technique of optical coherence tomography (OCT) instruments, images of the choroid have improved, making it possible to measure choroidal thickness more accurately, safely, and simply [9–13]. Several researchers have found that in high myopic eyes, the choroidal thickness is significantly thinner than in normal eyes [14–17]. However, most of these studies failed to adjust for potential compounding factors such as axial length (AL) and spherical equivalent (SE), which have been proven to have a negative correlation with choroidal thickness [18, 19].

Therefore, the aim of this study was to evaluate the macular choroidal thickness in Chinese patients with high myopia and normal subjects and to determine the correlation with clinical factors in high myopic eyes. In addition, because of the myopic CNV and the lacquer crack, which would develop into secondary chorioretinal central atrophy, we compare the subfoveal choroidal thickness (SFCT) in high myopic eyes with or without CNV and with or without lacquer cracks. Finally, given the data accumulated, we sought to conduct a comprehensive meta-analysis to calculate the differences in SFCT between high myopic eyes and normal control eyes quantificationally.

## Methods

### Subjects and enrollment criteria

This study was approved by the Ethical Review Committee of Aier Eye Hospital and adhered to the provisions of the Declaration of Helsinki for research involving human subjects. Written informed consent was obtained from the participants before the study began. All the subjects were from a Chinese Han population, and they were prospectively and consecutively recruited for this study between October 2012 and November 2014.

In the high myopia group, the inclusion criteria were as follows: (1) high myopia was defined as an SE > −6 diopters (D); (2) all eyes had clear ocular media; (3) a clear image was obtained to enable precise measurement of the choroidal thickness. Patients were excluded from this study if they presented with any retinal abnormalities other than high myopia such as diabetic retinopathy, uveitis, drusen, retinal vascular abnormalities, age-related macular degeneration, or other eye diseases such as the history of amblyopia or glaucoma. Patients who had a history of intraocular surgery, refractive surgery, or intravitreal injection were also excluded from the study. Patients with systemic diseases were also excluded. In the control group, the inclusion criterion was healthy eyes with an SE between −3 D to 3 D.

### Examination

All subjects underwent a refractive error examination without pupil dilation using autorefraction (NIDEK, AR-310A, Japan). SE was calculated as the sum of the spherical power and half of the cylinder power [20]. All the eyes of the subjects also underwent a thorough ophthalmic evaluation, including intraocular pressure (IOP) measurement, slit-lamp biomicroscopy, dilated ophthalmoscopy fundus examination, and color fundus photographs (Canon, Retinal Camera CR-DGi, Japan). B-scanning was also performed. AL measurements were taken using partial optical coherence interferometry (IOL-Master; Carl Zeiss Meditec, La Jolla, CA, USA). All the repeat measurements used the median for the analysis. Demographic data on each subject’s age and sex were collected.

### EDI-OCT examination

All subjects were examined with a commercial OCT machine with an EDI mode (RS-3000 SD-OCT; Nidek, Japan). Choroidal imaging was performed as described in a previous study [10]. In brief, the vertical and horizontal sections going directly through the centre of the fovea were used for final analysis. Choroidal thickness was defined as the distance between the retinal pigment epithelium (outermost hyperreflective line) to the inner margin of the sclera. Choroidal thicknesses were measured at the subfovea, 1 mm and 3 mm, nasally, temporally, superiorly, and inferiorly. All measurements were taken by a single experienced ophthalmologist who was masked to the clinical diagnosis of the subjects and was not involved in the data analysis. The images were obtained with the best visualization of the border between the choroid and the sclera known as the choroidal–scleral interface (CSI). If neither image had a clearly identifiable CSI, additional images were taken to produce the best possible view of the CSI. To avoid possible diurnal variation in choroidal thickness [21], all EDI-OCT examinations were performed between 9 AM and 12 AM.

### Statistical analysis

Statistical analysis was performed with SPSS software package version 17 (SPSS Inc, Chicago, IL, USA). Categorical covariates were assessed individually with the chi-square test. The demographics and ocular parameters between highly myopic and control eyes were compared using independent *t*-tests. One-way analysis of variance with the Bonferroni post-test was used to calculate the difference between the mean choroidal thicknesses of different locations. Univariate and multivariate regression analysis was used to evaluate the potential factors associated with SFCT in high myopia subjects. Univariate regression analyses were performed separately for each variable. Variables with a probability value ≤0.10 in univariate analyses were included in the multivariate analysis using a stepwise method. In high myopic eyes, subgroup analysis of macular choroidal thickness was performed in eyes with or without lacquer cracks and CNV using an independent-sample *t* test. For all the tests, *P* < 0.05 was considered significant.

### Meta-analysis

Literature searches were performed in the following databases: PubMed, ISI Web of Science, EMBASE in English and in the Chinese National Knowledge Infrastructure (CNKI) (http://www.cnki.net/), VIP (http://www.cqvip.com/), and Wan Fang (http://www.wanfangdata.com.cn) in Chinese. The following search strategy was performed in PubMed: ((Choroidal[All Fields] AND thickness[All Fields]) OR ("choroid"[MeSH Terms] OR "choroid"[All Fields])) AND (("myopia"[MeSH Terms] OR "myopia"[All Fields]) OR ("myopia"[MeSH Terms] OR "myopia"[All Fields] OR "myopic"[All Fields])). The search results were supplemented by reviews of reference lists for all relevant studies and review articles. If there were several studies published by the same population, the recent study was included. The final literature search was updated December 2014, with no restrictions on publication year, language, or methodological filter.

Studies that met the following criteria were included in this meta-analysis: (a) cross-sectional or case–control design; (b) choroidal thickness measured by OCT; (c) differences in choroidal thickness between patients with high myopia and controls reported. The exclusion criteria were as follows: (a) duplicate data; (b) no control population; (c) abstracts, comments, letters, case report, reviews, or editorial articles; (d) insufficient data on choroidal thickness. Two observers independently extracted the following information from the included studies, using a standardized data extraction form: first author, year of publication, number of high myopia patients and controls, definition of high myopia, the SE criteria of control subjects, and differences in choroidal thickness. Any discrepancies were addressed by having a discussion to reach a consensus.

To arrive at a conservative estimate of the effect of potential population differences among the studies, we chose a random-effect model to calculate pooled weighted mean differences (WMDs) in the meta-analysis. Statistical heterogeneity among the studies was evaluated using Cochran’s Q test and the I^2^ statistic. For the Q statistic, *P* < 0.05 was considered to indicate statistically significant heterogeneity [22, 23]. To explore the source of heterogeneity, subgroup analyses were done according to the instrument used, the mean age of included subjects, and the source of control. To evaluate the influence of an individual data set on the pooled results, one study was deleted at a time, and the combined estimates were recalculated based on the remaining studies. Potential publication bias was evaluated using funnel plots, Begg’s test, and Egger’s test [24, 25]. A *P* value less than 0.05 was considered statistically significant in the test results for overall effect. The meta-analyses were conducted using the Stata software package (Version 12.0; Stata Corp., College Station, TX).

## Results

### Demographic and baseline characteristics of the subjects

A total of 314 eyes of 178 highly myopic patients and 173 eyes of 109 normal participants were included in the study. Of these, 13 high myopic eyes and eight control eyes were excluded because the border between the choroid and the sclera could not be visualized, although the optical media was clear. Therefore, data from 301 eyes of 171 high myopic patients and 165 eyes of 103 normal participants with high-quality OCT images were available and included in the analysis. The mean age was 22.23 ± 6.50 years in high myopia patients and 23.36 ± 7.40 years in control subjects. The mean SEs for the high myopia group and the control group were −7.56 ± 1.99 D and −0.74 ± 1.47 D, respectively. The mean ALs were 26.56 ± 1.01 mm and 23.71 ± 0.89 mm in the high myopia and control groups, respectively. The demographic and baseline characteristics of the patients are summarized in Table 1. There was a significant difference between the two groups in SE (*P* < 0.001) and AL (*P* < 0.001). The two groups did not differ significantly in terms of age, sex, IOP, central corneal thickness (CCT), diastolic blood pressure, systolic blood pressure, diastolic ocular perfusion pressure, systolic ocular perfusion pressure, or mean ocular perfusion pressure.

**Table 1:** Clinical characteristics in study subjects

	High myopia	Normal Control	*P*
No. of patients (No. of eyes)	171 (301)	103 (165)	-
Mean age (SD), y	22.23 (6.50)	23.36 (7.40)	0.088
Gender (male/female)	119/52	66/37	0.345
IOP at imaging (SD), mm Hg	14.56 (3.66)	14.84 (3.52)	0.429
Spherical equivalent (SD), D	−7.56 (1.99)	−0.74 (1.47)	<0.001
Axial length (SD), mm	26.56 (1.01)	23.71 (0.89)	<0.001
CCT, μm	544.61 (33.23)	541.44 (33.63)	0.328
DBP, mmHg (SD)	74.79(8.55)	75.26(8.94)	0.577
SBP, mmHg (SD)	120.29(11.65)	122.53(13.81)	0.064
Diastolic OPP, mmHg (SD)^1^	60.23(9.04)	60.42(9.62)	0.829
Systolic OPP, mmHg (SD)^2^	105.73(12.18)	107.69(14.03)	0.116
Mean OPP, mmHg (SD)^3^	75.40 (8.87)	76.18(9.30)	0.369

*SD* standard deviation, *IOP* intraocular pressure, *D* diopter, *CCT* central corneal thickness, *DBP* diastolic blood pressure, *SBP* systolic blood pressure, *OPP* ocular perfusion pressure

^1,2^Calculated as the differential pressure between diastolic or systolic blood pressure and IOP

^3^Calculated as the differential pressure between mean BP and IOP (mean BP = DBP + 1/3*(SBP -DBP))

### Choroidal thickness in the macular region

The choroidal thickness data at the fovea and 1 mm and 3 mm from the center temporally, nasally, superiorly, and inferiorly are shown in Table 2. The high myopia group (200.54 ± 69.39 μm) exhibited a significantly thinner subfoveal choroid than the control group (276.21 ± 64.67 μm; *P* < 0.001). The macular choroidal thickness was significantly lower in the high myopia group than in the control group at all locations (*P* < 0.001).

**Table 2:** Average choroidal thickness and 95 % CI at different locations in macula

Location (mm from fovea)	High myopia		Control		Mean Difference (μm)^a^	95%CI (μm)		*P*
	Mean (μm)	SD (μm)	Mean (μm)	SD (μm)		Lower Bound	Upper Bound	
SFCT	200.54	69.39	276.21	64.67	−75.67	−88.56	−62.77	<0.001
Superior 1 mm	215.06	61.30	251.88	68.44	−36.81	−49.04	−24.57	<0.001
Superior 3 mm	197.71	63.96	257.91	64.64	−60.19	−72.54	−47.85	<0.001
Inferior 1 mm	198.90	61.66	242.11	73.16	−43.21	−56.51	−29.91	<0.001
Inferior 3 mm	182.56	59.60	227.67	76.01	−45.11	−57.71	−32.51	<0.001
Nasal 1 mm	171.76	66.11	226.07	76.26	−54.30	−67.60	−41.00	<0.001
Nasal 3 mm	104.90	45.97	150.10	62.27	−45.20	−55.16	−35.24	<0.001
Temporal 1 mm	189.29	67.02	250.52	69.04	−61.22	−74.12	−48.33	<0.001
Temporal 3 mm	181.58	65.64	242.04	72.16	−60.46	−73.78	−47.13	<0.001

*CI* confidence interval, *SD* standard deviation, *SFCT* subfoveal choroidal thickness

^a^Normal group as reference

### Univariate and multiple linear regression analysis

Univariate and multivariate regression analyses were performed to determine the factors associated with SFCT in all subjects. Univariate regression analyses showed that diagnosis, SE, and AL factors were associated significantly with SFCT. In this univariate regression analyses, AL accounted for 31.8 %, SE explained 26.1 %, and diagnosis explained 22.1 % of variation in choroid thickness. We then performed stepwise multiple linear analysis to determine the factors associated with SFCT in both groups. The model included the diagnosis, ES, and AL. After adjusting for the AL and ES by multivariate linear regression analysis, a diagnosis was not associated with SFCT (*P* =0.471). Table 3 shows the detailed results of the linear regression analysis.

**Table 3:** Association between SFCT with other factors

Factor	Beta (95 % CI)	Adjusted R^2^	*P*
Univariate analysis			
Diagnosis (high myopia vs normal control)	−75.67(−88.57, −62.77)	0.221	<0.001
Age, year	0.86 (−0.17, 1.88)	0.004	0.100
Gender (male/female)	−6.85 (−21.95, 8.25)	0	0.373
IOP at imaging, mm Hg	−0.28 (−2.22, 1.67)	0.002	0.781
Spherical equivalent, D	10.52 (8.91, 12.13)	0.261	<0.001
Axial length, mm	−26.02 (−29.48, −22.55)	0.318	<0.001
CCT, μm	−0.16 (−0.40, 0.08)	0.006	0.179
DBP, mmHg	0.66 (−0.14, 1.47)	0.003	0.106
SBP, mmHg	0.41 (−0.15, 0.97)	0.002	0.147
Diastolic OPP, mmHg	0.63 (−0.13, 1.38)	0.004	0.104
Systolic OPP, mmHg	0.41 (−0.13, 0.95)	0.003	0.138
Mean OPP, mmHg	0.72 (−0.06, 1.49)	0.005	0.069
Multivariate analysis			
Diagnosis (high myopia vs normal control)	−9.55 (−35.57, 16.48)	-	0.471
Spherical equivalent (D)	3.36 (−0.21, 6.92)	-	0.065
Axial length (mm)	−21.93 (−28.65, −15.22)	-	<0.001

*SFCT* subfoveal choroidal thickness, *CI* confidence interval *IOP* intraocular pressure, *D* diopter, *CCT* central corneal thickness, *DBP* diastolic blood pressure, *SBP* systolic blood pressure, *OPP* ocular Perfusion Pressure

### Subgroup analysis of high myopia group

To test whether patient with CNV or lacquer cracks in the high myopia group affected SFCT, the SFCT levels were compared between the patients with CNV and the patients without CNV. Table 4 summarizes the baseline characteristics of these two subgroups. The patients with CNV had a larger SE and longer AL than those without CNV. There were no significant differences in other variables between the two subgroups. When we compared the SFCT between the two subgroups, we found that the high myopic eyes with CNV had a significant thinner choroid than high myopic eyes without CNV. We also divided the high myopic eyes into two subgroups based on their appearance of lacquer cracks. The baseline characteristics of the lacquer crack subgroup also had a larger SE and longer AL than the subgroup without lacquer cracks, and other variables were comparable between the two subgroups. When comparing the SFCT between the two subgroups, we also found that the high myopic eyes with lacquer cracks had a significantly thinner choroid than the high myopic eyes without lacquer cracks.

**Table 4:** Clinical characteristics in different high myopia subgroup

Variables	With or without Lacquer Cracks subgroup		*P*	With or without CNV subgroup		*P*
	Lacquer Cracks (*n* =32)	No Lacquer Cracks (*n* =269)		CNV (*n* =21)	No CNV (*n* =280)	
Mean age (SD), y	24.94 (8.15)	21.91 (6.21)	0.050	24.86(8.53)	22.04 (6.29)	0.152
Gender (male/female)	22/10	189/80	0.860	13/8	198/82	0.395
IOP at imaging (SD), mm Hg	15.63 (3.51)	14.43 (3.65)	0.077	15.10 (3.80)	14.52 (3.65)	0.487
Spherical equivalent (SD), D	−10.25 (2.63)	−7.23 (1.62)	<0.001	−10.66 (2.10)	−7.32 (1.77)	<0.001
Axial length (SD), mm	27.42 (1.41)	26.44 (0.90)	<0.001	27.43 (1.23)	26.48 (0.96)	<0.001
CCT, μm	554.15 (37.42)	543.47 (32.58)	0.131	546.38 (41.53)	544.47 (32.61)	0.800
DBP, mmHg (SD)	74.43 (7.40)	74.83 (8.68)	0.781	73.57 (8.15)	74.88 (8.58)	0.499
SBP, mmHg (SD)	119.87 (11.47)	120.33 (11.68)	0.832	118.57 (8.55)	120.41 (11.84)	0.485
Diastolic OPP, mmHg (SD)^1^	58.05 (7.88)	60.49 (9.15)	0.149	58.00 (7.72)	60.39 (9.12)	0.242
Systolic OPP, mmHg (SD)^2^	103.48 (12.14)	105.99 (12.18)	0.272	103.00 (7.41)	105.93 (12.45)	0.288
Mean OPP, mmHg (SD)^3^	73.19 (7.69)	75.65 (8.97)	0.138	72.99 (6.44)	75.58 (9.01)	0.199
SFCT	145.03 (73.53)	207.14 (65.97)	<0.001	144.14 (54.54)	204.77 (68.61)	<0.001

*CNV* Choroidal neovascularization, *SD* standard deviation, *IOP* intraocular pressure, *D* Diopter, *CCT* central corneal thickness, *DBP* diastolic blood pressure, *SBP* systolic blood pressure, *OPP* ocular perfusion pressure, *SFCT* subfoveal choroidal thickness

^1,2^Calculated as the differential pressure between Diastolic or Systolic Blood Pressure and IOP

^3^Calculated as the differential pressure between mean BP and IOP (mean BP = DBP + 1/3*(SBP -DBP))

### Factors associated with SFCT in high myopic eyes

Univariate and multivariate regression analyses were performed to determine the factors associated with SFCT in high myopic eyes. The variables associated significantly with SFCT by univariate regression were SE, AL, with CNV, and with lacquer cracks. These associative variables were then entered into a multivariate regression analysis. Independent factors of choroidal thinning were AL, the presence of CNV, and the presence of lacquer cracks (all *P* < 0.05). The results of these analyses are shown in Table 5.

**Table 5:** Association of factors with SFCT in highly myopic eyes

Factor	Beta (95 % CI)	*P*
Univariate analysis		
Age, year	0.40 (−0.83, 1.60)	0.529
Gender (male/female)	−2.43 (−19.64, 14.79)	0.782
IOP at imaging, mm Hg	−1.29 (−3.45, 0.86)	0.239
Spherical equivalent, D	10.21 (6.41, 14.02)	<0.001
Axial length, mm	−31.90 (−38.86, −24.95)	<0.001
CCT, μm	−0.16 (−0.40, 0.08)	0.179
DBP, mmHg	1.34 (−0.43, 2.25)	0.112
SBP, mmHg	0.50 (−0.18, 1.18)	0.147
Diastolic OPP, mmHg	1.47 (0.61, 2.33)	0.079
Systolic OPP, mmHg	0.61 (−0.04, 1.25)	0.064
Mean OPP, mmHg	1.40 (−0.53, 2.28)	0.067
Lacquer cracks (yes/no)	−62.12 (−86.70, −37.54)	<0.001
CNV (yes/no)	−60.63 (−90.80, −30.46)	<0.001
Multivariate analysis		
Axial length, mm	−30.87 (−38.15, −23.59)	<0.001
Lacquer Cracks (yes/no)	−28.76 (−51.84, −5.67)	0.015
CNV (yes/no)	−17.98 (−33.25, −2.73)	0.021

*SFCT* subfoveal choroidal thickness, *CI* confidence interval, *IOP* intraocular pressure, *D* diopter, *CCT* central corneal thickness, *DBP* diastolic blood pressure, *SBP* systolic blood pressure, *OPP* ocular Perfusion Pressure, *CNV* choroidal neovascularization

### Eligible articles for meta-analysis

**Fig. 1:**
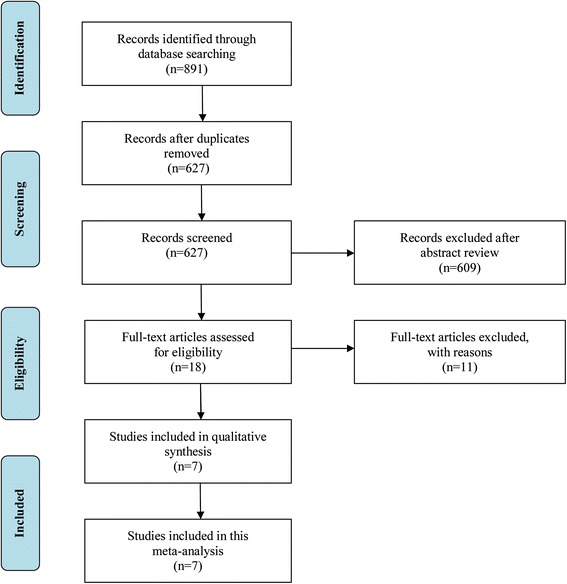
Flow diagram outlining the selection process for inclusion of studies in the systematic review and meta-analysis

To obtain more information about the difference in choroidal thickness between the highly myopic and normal control eyes, we performed a meta-analysis. The initial search yielded 891 potentially relevant studies. After the removal of duplicates found in the electronic databases, 627 studies remained. Based on their titles and abstracts, 609 articles were excluded because of their apparent irrelevance. Eighteen full-text articles were further assessed for eligibility. Finally, seven articles met the inclusion criteria and were included in this meta-analysis [14–17, 26–28]. Of these seven eligible articles, four were written in English [14–17], and three in Chinese [26–28]. The study selection process is shown in detail in Fig. 1.

### Characteristics of the included studies

Overall, the seven studies plus the present study with 1204/630 high myopic eyes/control eyes were included in this meta-analysis. Among these studies, five originated from China, one from Spain, one from Singapore, and one from Japan. The used examination instrument included Heidelberg Spectralis, Topcon 3D-2000, Zeiss Cirrus, and Nidek RS-3000. In all included studies, the definition and the included high myopic eyes were the SE > −6 D. However, the SE of the control eyes included in different studies was not uniform and ranged from emmetropia to ± 6 D. The main characteristics of the included studies are presented in Table 6.

**Table 6:** Characteristics of included studies

First Author (year)	Location	CT	Instrument	Age (year)	Definition of high myopia	The SE criteria of control subjects	No eyes (High myoipa/control)
Flores-Moreno (2013) [16]	Spain	SFCT	Topcon 3D-2000	54.4/52.6	SE > −6.0 D	−6 D to +6 D	120/96
Ohsugi (2013) [17]	Japan	SFCT	Heidelberg Spectralis	65.4/67.2	SE > −6.0 D	−3 D to +3 D	25/25
Mo (2013) [28]	China	SFCT	Heidelberg Spectralis	29.27/26.37	SE > −6.0 D	Emmetropia	96/72
Gupta (2014) [14]	Singapore	SFCT	Heidelberg Spectralis	21.59/22.06	SE > −6.0 D	<0.5 D	520/128
Chen (2014) [15]	China	SFCT	Zeiss Cirrus	59.6/58.5	SE > −6.0 D	−3 D to +3 D	36/42
Qi (2014) [27]	China	SFCT	Heidelberg Spectralis	33.51/34.70	SE > −6.0 D	−2 D to +2 D	75/70
Chen (2014) [15]	China	SFCT	Zeiss Cirrus	29.1/29.6	SE > −6.0 D	Emmetropia	31/32
Present (2014)	China	SFCT	Nidek RS-3000	22.23/23.36	SE > −6.0 D	−3 D to +3 D	301/165

*CT* choroidal thickness, *SE* spherical equivalent, *SFCT* subfoveal choroidal thickness, *D* diopter

### Meta-analysis results

In the comparison of the SFCT of high myopic eyes with the control eyes, the pooled WMDs were calculated using the random-effect model. The meta-analysis of these data showed that the SFCT was significantly thinner in the high myopic eyes than in the controls (WMD = −116.30 μm; 95 % CI: −145.68, −86.92; *P* < 0.001). Significant heterogeneity was observed between studies. Considering that the precision of the OCT was 10 μm, this difference could have been caused by instrument error. We then stratified the studies based on the instrument used. Subgroup analysis showed that a different examination instrument obtained similar results. In addition, the previous study had proved that choroidal thickness was negative association with age [19]. In the present meta-analysis, the mean age of included subjects in different studies ranged from 21.6 to 67.2 years, which might affect the pooled results. Therefore, we also stratified the studies according to the mean age of included subjects (>50 years or < 50 years). Both subgroups also showed a similar result in that the SFCT of high myopic eyes was significantly thinner than the control eyes. However, as expected, the > 50 years subgroup showed the larger WMD than the < 50 years subgroup. The detailed results are shown in Table 7.

**Table 7:** Pooled estimates of all studies comparing SFCT in high myopic eyes with normal control eyes

	No. of studies	WMD (random)(95 % CI)	Test for Heterogeneity	Test for Overall Effect
Instrument				
All trials	8	−116.30 (−145.68, −86.92)	Q = 108.64, *P* <0.001	Z = 7.76, *P* <0.001
Heidelberg Spectralis	4	−141.03 (−159.43, −122.63)	Q =75.46, *P* <0.001	Z = 15.02, *P* <0.001
Zeiss Cirrus	2	−80.36 (−155.77, −4.95)	Q = 17.43, *P* <0.001	Z =2.09, *P* =0.037
Topcon 3D-2000	1	−142.40 (−167.45, −117.35)	-	Z =11.14, *P* <0.001
Nidek RS-3000	1	−75.67 (−88.27, −63.07)	-	Z =11.77, *P* <0.001
The mean age of included subjects				
All trials	8	−116.30 (−145.68, −86.92)	Q = 108.64, *P* <0.001	Z = 7.76, *P* <0.001
> 50 years	3	−128.19 (−143.37, −113.01)	Q =1.98, *P* <0.001	Z = 16.55, *P* <0.001
< 50 years	5	−109.56 (−152.64, −66.47)	Q =101.70, *P* <0.001	Z =4.98, *P* <0.001

*SFCT* subfoveal choroidal thickness, *WMD* weighted mean differences, *CI* confidence interval

### Sensitivity analysis and publication bias

To evaluate the influence of an individual data set on the pooled results, one study was deleted at a time. The corresponding estimates did not change greatly when any single study was deleted (Table 8).Publication bias was tested using Begg’s test (*P* = 0.902) and Egger’s test (*P* = 0.616), and no obvious evidence of publication bias was found.

**Table 8:** Sensitivity analysis of the meta-analysis

	Random effects model		Test of homogeneity		
Study Excluded	WMD	95 % CI	Q	I^2^ (%)	*P* value
None	−116.30	−145.68, −86.92	108.64	93.6	<0.001
Flores-Moreno (2013) [16]	−112.60	−145.01, −80.20	102.87	94.2	<0.001
Ohsugi (2013) [17]	−115.51	−148.03, −82.99	108.29	94.5	<0.001
Mo (2013) [28]	−115.95	−149.34, −82.56	108.41	94.5	<0.001
Gupta (2014) [14]	−111.37	−143.17, −79.56	86.80	93.1	<0.001
Chen (2014) [15]	−115.85	−148.61, −83.08	108.43	94.5	<0.001
Qi (2014) [27]	−109.84	−139.86, −79.82	80.81	92.6	<0.001
Chen (2014) [15]	−126.65	−154.96, −98.35	79.35	92.4	<0.001
Present (2014, unpublished)	−122.68	−150.54, −94.82	59.02	89.8	<0.001

*WMD* weighted mean difference, *CI* confidence Interval

## Discussion

In this cross-sectional study, we measured the macular choroidal thickness in high myopic eyes and normal control eyes. The mean SFCT of the high myopia group in this study was 200.54 ± 69.39 μm. Compared with the normal eyes, the results of this study revealed that the choroidal thickness was significantly smaller in the high myopic eyes. A thinning of the choroid in high myopic eyes has been demonstrated in several other studies [14–17]. More importantly, these findings are supported by the results of the present meta-analysis, which showed that the SFCT of high myopic eyes was significantly thinner than that of the normal control eyes with the pooled WMD of −116.30 μm (95 % CI: −145.68, −86.92). This was not surprising. It has been reported that in high myopic eyes, excessive axial elongation of the eyeballs can cause biomechanical stretching and thinning of the choroid, retina, and sclera [29, 30]. Thus, the thinner choroid is a long-term correlate of myopic axial elongation of the eye. In their previous studies, Gupta et al. [14] found that high myopic eyes have significantly thinner choroid than that of emmetropic eyes. However, they failed to adjust for relative compounding factors such as AL and SE, and we do not know whether the thinning choroid is because of the longer AL or an independent factor. Chen et al. [15] and Ohsugi et al. [17] both obtained results similar to those of Gupta et al. However, they also failed to adjust for any potential compounding factors that might affect the choroidal thickness such as age, AL, and SE. Thus, in the present study, we investigated whether the thinner choroid in high myopic eyes might be due to longer AL and larger SE. Surprisingly, after adjusting for the AL and SE by multivariate linear regression analysis, we found that the diagnosis was not associated with SFCT. Therefore, we concluded that the thinner SFCT in high myopic eyes might be secondary in its effects to the longer AL, but it is not an independent factor.

In this study, we also performed a meta-analysis to calculate the difference in the SFCT between high myopic eyes and normal control eyes quantificationally. After a systematic literature search of several databases, we reviewed seven studies, including ours, to compare the SFCT in high myopic eyes with that of normal control eyes. Our findings of significance were also reflected in the meta-analysis, even across different subgroups, which are less prone to chance results, indicating the robustness of our findings. It is also worth mentioning that the "leave-one-out" sensitivity analyses did not affect the pooled results of the meta-analysis, which illustrated that the present meta-analysis results were stable and reliable.

The formation of lacquer cracks is considered a risk factor for developing CNV [31, 32]. A previous study reported that the high myopic eyes with lacquer cracks had a less choroidal thickness than those without lacquer cracks [33]. El and associates [34] reported that macular choroidal thinning was also observed in high myopic eyes with CNV. In order to test whether the presence of CNV or lacquer cracks in high myopic eyes affected the SFCT, based on the relative large sample size, we divided the high myopic eyes into subgroups according to the presence or absence of CNV and lacquer cracks. The results showed that the subgroups with CNV and with lacquer cracks both had longer AL, larger SE, and more importantly, a thinner choroid. In order to determine whether the thinner choroid is secondary to the longer AL or the larger SE, we performed the univariate and multivariate linear regression analysis again. In the univariable analysis, the results showed that SE, AL, the presence of lacquer cracks, and the presence of CNV were significantly associated with SFCT. After adjusting for the SE and AL by multivariate linear regression analysis, the mean SFCTs of the eyes with lacquer cracks and CNV were still significantly thinner than those where these were absent. This implies that the thinner choroid in the presence of CNV and the presence of lacquer cracks were not secondary to the longer AL or the larger SE but rather was an independent factor. This result might indirectly prove once again that myopic CNV and lacquer cracks could develop into secondary chorioretinal central atrophy. The SFCT could be a good indicator and predictor of the clinical significance and severity of the disease in these high myopic eyes.

Our results show that the average SFCT in high myopic eyes was 200.54 ± 69.39 μm, which is much thicker than previous findings indicating a mean SFCT of 111.1 ± 45.0 μm [15] and 115.0 ± 85.3 μm [16]. It was also significantly thicker than the pooled results of the present meta-analysis (−116.30 μm). Considering this difference, we speculated that participants’ ages might be the main cause. It has been proven that age is negatively associated with choroidal thickness. In our study, the mean age of high myopia is only 22.23 years, which is younger than in other studies [15–17]. Another important point is that, in all subjects, age was not associated with choroidal thickness, which is inconsistent with the findings of a large number of studies [19, 35, 36]. One possible explanation for this is that in our study, the age distribution of patients included is limited to a young age group; further studies including patients from a wide range of ages are needed to shed light on this difference. Another possible explanation is that other factors' strong association with the SFCT, such as AL and SE, concealed the factor of age. In the present study, we performed the EDI-OCT examination using the RS-3000 SD-OCT machine, which is different from the machines used in other studies. However, the results of the subgroup analysis showed that different examination instruments had similar results. This implied that using a different OCT machine would not affect the results. In addition, the previous studies proved that the choroidal thickness measurements obtained with different SD-OCTs were highly correlated and could be used interchangeably [37, 38].

Our study had a number of strengths. First, to date, the present study is the first synthesis exploring the association of choroidal thickness with high myopia. Second, the results of the present cross-sectional study were in accord with that of the corresponding meta-analysis. Third, our results are less prone to selection bias, in view of the low probability of publication bias.

It should be noted that the present study has a few limitations. First, in the meta-analysis, the SE of the control eyes included in the various studies was not uniform—it ranged from emmetropia to ± 6 D, which might affect the pooled results. Second, the small number of trials eligible for our meta-analysis made it difficult to acquire enough data to obtain meaningful results. Third, in the meta-analysis, substantial heterogeneity was observed among the studies. Although we performed analyses of the subgroups and sensitivity analyses, this heterogeneity could not be fully explained by the results. Fourth, the measurements of the choroidal thickness were performed manually, and automated software will be required for a more objective evaluation. However, the previous studies have proven that choroidal thickness measurement done using EDI-OCT is highly reproducible and repeatable [39, 40]. Finally, given that factors other than AL and SE likely affect the choroidal thickness, our conclusion should be viewed with caution.

## Conclusion

The present study, along with the comprehensive meta-analysis, indicated that in the Chinese population, the choroidal thickness in high myopic eyes was thinner than that of normal control eyes, even across different subgroups. This might be secondary to the longer AL but it is not an independent factor. The presence of CNV and of lacquer cracks is associated strongly with eyes with thinner macular choroids. The SFCT might be a good indicator of the severity of myopic maculopathy.
